# Path Loss Determination Using Linear and Cubic Regression Inside a Classic Tomato Greenhouse

**DOI:** 10.3390/ijerph16101744

**Published:** 2019-05-17

**Authors:** Dora Cama-Pinto, Miguel Damas, Juan Antonio Holgado-Terriza, Francisco Gómez-Mula, Alejandro Cama-Pinto

**Affiliations:** 1 Department of Computer Architecture and Technology, University of Granada, 18071 Granada, Spain; mdamas@ugr.es (M.D.); frgomez@ugr.es (F.G.-M.); 2 Software Engineering Department, University of Granada, 18071 Granada, Spain; jholgado@ugr.es; 3 Department of Computer Sciences and Electronic, Universidad de la Costa, Barranquilla 080002, Atlantico, Colombia; acama1@cuc.edu.co

**Keywords:** propagation model, wireless propagation model, precision agriculture, COST235, ITU-R, FITU-R, Weisbberger model

## Abstract

The production of tomatoes in greenhouses, in addition to its relevance in nutrition and health, is an activity of the agroindustry with high economic importance in Spain, the first exporter in Europe of this vegetable. The technological updating with precision agriculture, implemented in order to ensure adequate production, leads to a deployment planning of wireless sensors with limited coverage by the attenuation of radio waves in the presence of vegetation. The well-known propagation models FSPL (Free-Space Path Loss), two-ray, COST235, Weissberger, ITU-R (International Telecommunications Union—Radiocommunication Sector), FITU-R (Fitted ITU-R), offer values with an error percentage higher than 30% in the 2.4 GHz band in relation to those measured in field tests. As a substantial improvement, we have developed optimized propagation models, with an error estimate of less than 9% in the worst-case scenario for the later benefit of farmers, consumers and the economic chain in the production of tomatoes.

## 1. Introduction

The Food and Agriculture Organization of the United Nations (FAO) predicts that the world population will reach 8 billion people by 2025 and 9.6 billion by 2050 [1]. One of the most promising concepts, which is expected to contribute greatly to the necessary increase in food production in a sustainable manner, is precision agriculture (PA) [2,3]; a set of technologies used to understand changes during planting cycles. Precision agriculture arises from the need for technologies to collect information in agricultural areas about soil conditions from the environment and transmit data. It offers the means for agricultural practices to be monitored, evaluated and controlled. This information directly affects the decision-making on the activities to be carried out throughout the plantation.

The PA relies on applications of wireless sensor networks (WSN) in the function of monitoring and controlling the management of a field because it reduces the costs of monitoring and managing crop production at the plant level, instead of monitoring the entire greenhouse [4,5,6,7,8,9] or with the purpose to make environmental measurements (i.e., on water or soil) [10]. In PA, the deployment of WSN has provided greater financial returns by optimizing product quality and quantity of yield while minimizing costs [11,12,13,14]. Therefore, in this paper we focus on studying and comparing the phenomenon of propagation and attenuation of radio waves in the unlicensed band of 2425 MHz; the most commonly used in the world of precision agriculture [3] inside the tomato production greenhouse.

### Background

The surface of greenhouses in the world exceeds 700,000 ha, being concentrated mainly in Asia, in the Mediterranean basin, and in central and northern Europe [15]. In this order of ideas, world exports of tomatoes exceeded 6.9 billion euros in 2013, with the EU, the main sector of world trade in fresh tomatoes, with 57.8% in 2013 (volume in kg) and 65.7% of turnover (volume in euros). Spain is the main supplier, with 23.78% and 21.7% of EU imports in tons and euros respectively. Of the 27,000 ha of greenhouses in the province of Almería, 10,232 ha are destined to fresh tomato crops, the main crop in the greenhouses of Almería with a total production of 958,462 tons, which represents 83.2% of the surface and 61% of the total production of Andalusia having the first place in the national production. Regarding exports, its main markets are in the EU, especially Germany, France, the Netherlands and the United Kingdom [16,17]. Due to this relevance, our research is focused on tomato greenhouses. Accordingly, the most widespread empirical propagation models in the presence of vegetation are those shown in Table 1. However, because they are general, the predictions that the aforementioned models show could be improved with values closer to the real ones, helping to contribute to better planning in the deployment being this the main object of our study.

According to the literature reviewed we have found similar research that studies the influence of foliage on radio path losses for WSN planning orchards [18] and a study that improves the path loss model for wireless sensor networks in mango greenhouses [19], others based on deployment of WSN over tomato greenhouses for monitoring environmental variables [20], and precision agriculture [21], but none of the reviewed literature was related to radio wave propagation models for tomato production greenhouse as in our work.

## 2. Materials and Methods

### 2.1. The Wireless Sensor Network

Wireless sensor networks (WSN) are one of the most important technologies of the 21st century [22] being an optimized form of acquisition and transmission of information [23], overcoming deployment difficulties and high installation and maintenance costs [24]. Wirelessly, a network can be quickly built automatically, using hierarchical network communication protocols and distributed algorithms. On the other hand, given that the nodes of the sensors are small in size and have a good capacity for cooperation, their deployment has a small impact on the agricultural environment. They also have other advantages, such as low energy consumption, self-organization capacity and local processing, constituting a promising platform for the implementation of monitoring systems that are increasingly used in agriculture [25,26,27,28,29,30].

The WSN in our study is based on the IEEE 802.15.4 standard, operating in the 2.4 GHz band, has lower width of the Fresnel zones compared to the 868, 915 MHz bands and with a faster transmission speed [6]. It is an important tool for environmental monitoring [31,32], and its use in rural areas and in the presence of vegetation is growing exponentially [33,34]. It establishes communication and detection infrastructures in areas where, otherwise, it would be impractical or impossible to do so [35]. However, each sensor used in the WSN has a limited scope [36]. Therefore, to efficiently plan and deploy WSNs in the presence of vegetation, it is crucial to have knowledge of the position, the level of transmission power and the propagation of the radio signal in the deployment environment [37]. The attenuation of the propagated signal increases with distance [38], and in areas of dense vegetation, such as orchards and forests, where the line of sight (LOS) between the nodes is typically non-existent, the foliage of trees may cause additional attenuation [39] by diffraction, reflection and scattering if there is no line of sight (NLOS) [40], especially due to the presence of water within the leaves and stems [41].

### 2.2. Received Signal Strength Indicator(RSSI)

The intensity of the received signal (RSSI—received signal strength indicator) is used to know the propagation of the radio wave [42,43,44]. The surrounding environment, the growth of crops and the different antenna heights will influence the measurement of RSSI [37]. In that sense, the models used to predict the RSSI between two transceivers are called propagation models [45].

### 2.3. Propagation with Line of Sight

1. Loss in free space. When an electromagnetic wave (EM) propagates in free space, path loss can be calculated using the Friis equation, widely used by microwave link designers [42], which assumes the absence of obstacles in the vicinity [39,46]: $\frac{P_{t}}{P_{r}}={\left(\frac{4\pi fd}{c}\right)}^{2}$, where Pr and Pt are the receiver and transmitter power respectively, *f* is the frequency of the radiation wave, *c*, the speed of light in the vacuum and *d* is the distance between the transmitter and the receiver [36,47]. The loss of the path in the free space means that the transceiver antennas, both transmitters and receivers, use communication with LOS, without obstructions or reflections of any kind. However, if the antennas are located close to the ground, the above equation is no longer valid, the reflection of the earth must be taken into account [18]. The power loss is usually expressed in terms of “path loss” (PL), defined as: PL = 10log10(P_t_/P_r_) [48] with P_t_ and P_r_ as power transmitted and received, respectively. Therefore, PL in free space can be expressed: PL_Free-space_(dB) = 20log(*f*) + 20log(*d*) − 147.56, the radiofrequency, *f*, is expressed in Hz and distance is expressed in meters [49,50].
2. Two-ray propagation model. When the RF propagates near the ground with LOS, the flat ground wave (PE) propagation model can be used to define the path loss instead of the PL_Free-space_ model. This model includes the effects of the reflection of the rays of the ground and the ray LOS, which is given by the equation: PL_PE_(dB) = 40log(*d*) − 20log(*h_T_*) − 20log(*h_R_*), where *d* is the distance between the transmitter and receiver antennas in meters, *h_T_* and *h_R_* are the elevations of the transceiver antennas in meters. The separation distance (*d*) in this model is much greater than *h_T_* and *h_R_* [45].

### 2.4. Total Loss

Signals at millimeter wave and microwave frequencies experience scattering and absorption caused by leaves and branches of vegetation randomly distributed [51]. Therefore, the total path losses are formulated by combining the losses of the PL_Free-space_ model with the PL_veg_ vegetation losses that are predicted by the different vegetation models: PL_tot_ = PL_Free-space_ + PE_veg_, where PL_tot_ is the total path loss [4].

### 2.5. Link Budget

The link budget is used to obtain the signal strength in the receiver, considering all the losses in the path between the transmitter and the receiver. The received power, which represents all the gains and losses is defined by the equation: P_r_(dBm) = P_t_ + G_t_ + G_r_ − L_path_, where P_r_ and P_t_ are the power received and transmitted. G_t_ and G_r_ are the gains of the transmitter and receiver; L_path_ is the total path loss [5].

### 2.6. Propagation with Non Line of Sight (NLOS)

**Figure 1:**
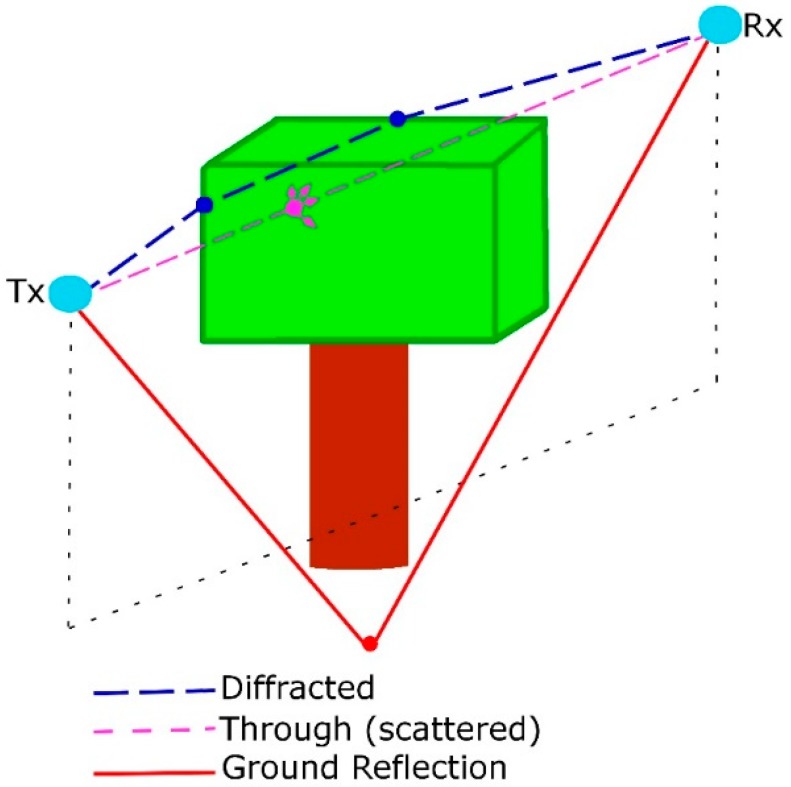
Possible mechanisms of propagation in the presence of vegetation.

It is important to study the effects of vegetation on a signal because many applications require the use of the microwave frequency band (0.3–300 GHz). In addition, depending on the thickness of the vegetation and the frequency of operation, a signal could travel along diffracted trails reducing the range of radio equipment communications. Quantitative knowledge of the excess of propagation loss suffered by radio waves due to the presence of vegetation is essential to plan a communication link in any wooded land [52,53,54,55]. Inside a greenhouse, in addition to attenuation in free space, electromagnetic waves are vanished by mechanisms that include diffraction, reflection, and dispersion produced by the leaves, branches, and stems of crops, distributed at random, between the transmitter and receiver, having an unknown effect on the exact propagation of radio waves (see Figure 1) [56]. To determine these changes, propagation models are used that estimate the radio coverage area of a transmitter and show the strength of the signal between the transmitter and receiver [11,12,36,57].

### 2.7. Propagation Model

Propagation models to predict the excess attenuation produced by vegetation were developed to model the excess attenuation found in a forest beyond what is predicted by free space or two-ray propagation [58]. These models can be theoretical and empirical. Theorists or analysts require a large database of environmental characteristics, requiring knowledge of parameters such as electromagnetic, soil moisture and leaves, geometrical characteristics, etc., which may be impractical and therefore we do not use it in our investigation. On the other hand, the empirical path loss models are based on actual radio frequency (RF) measurements of wireless channels [19]. Their main advantages over theoretical path loss models is the simplicity of the mathematical expressions applied that facilitate their direct application, their ease of implementation and their ability to include a greater environment-related factors that affect the propagation of radio waves. However, they do not take into account the geometry of the site [25,45,58,59,60]. Within the models of empirical propagation, there is the model of exponential decay proposed by Weissberger (1982), the model COST235 (1996) that considered the situation of trees with leaves and without it. Additionally to Recommendation ITU-R (CCIR 1986), Al-Nuaimi and Stephens (1998) proposed the FITU-R model. Each of the models used in the development of the research is summarized in Table 1 [61,62,63,64,65,66,67,68,69,70,71,72].

The Weissberger model expresses the excess attenuation (dB) of an obstacle in the propagation path. It is applicable in situations in which propagation is likely to occur through a grove and not by diffraction on the tree crown [37,66,72]. The basic model MED (Modified Exponential Decay) is described as $Att_{MED}=Xf^{y}d^{z}$, where *f* is the frequency in megahertz (MHz), *d* is the depth of the vegetation in meters and *X*, *Y* and *Z* are parameters adjusted by techniques of regression [59]. In that sense, the intensity of the received signal (RSSI) for wireless systems in vegetation media is largely based on empirical models that are relatively easy to use [73].

### 2.8. Hardware

**Figure 2:**
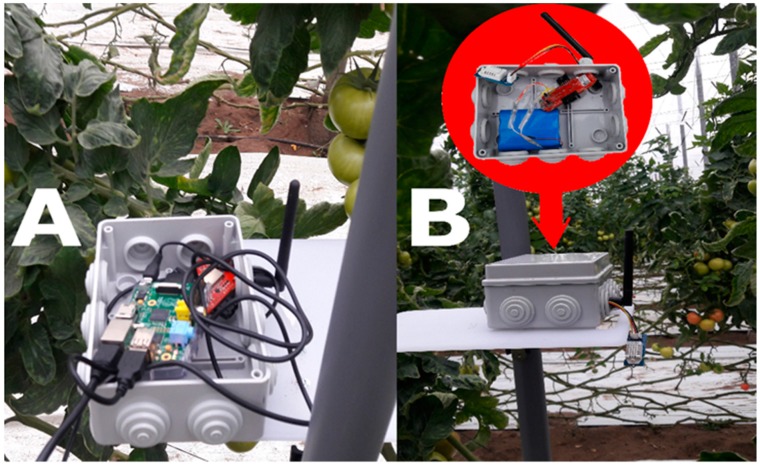
Wireless sensor networks (WSN) nodes. (**A**) Sink node connected to the Raspberry Pi. (**B**) Sensor node powered by an external lithium battery.

1. Raspberry Pi. The Raspberry Pi 3 computer receives data from the sensor node through the sink node connected to its USB port. The electric energy, available inside the greenhouse, fed the Raspberry Pi uninterruptedly during the testing stage.
2. Sensor and sink nodes. We used the Re-Mote nodes [74] that operate with the 2.4 GHz band (CC2538 System-on-Chip) for both the sensor node and the sink. The sink node is powered by the power provided by the USB cable connected to the Raspberry Pi computer (See Figure 2A), while the sensor node has a rechargeable Lithium-ion battery of 3.7 V and 6600 mAh (See Figure 2B).

### 2.9. Software

The Contiki operating system has been used in the wireless nodes. The applications were written in programming language C so that the sensor node sends information to the sink node, and it receives it and forwards it to the embedded computer (Raspberry Pi). The sensor node is programmed to save energy consumption so that its radio module at 2.4 GHz is not active all the time, giving greater durability to the lithium-ion battery while the sink node is constantly fed from the computer. The Raspberry Pi works with the Raspbian operating system, and through a script written in Python, it collects the information from the sink node in its USB port and then stores it in CSV format in its μSD memory.

### 2.10. Test Environment

**Figure 3:**
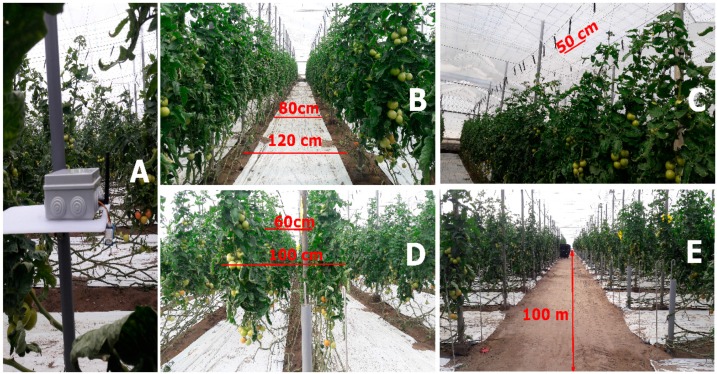
Internal view of the tomato greenhouse in Almeria. (**A**) Sensor node, (**B**) aisle dimensions, (**C**) distance between floors, (**D**) separation of paired lines, (**E**) main hallway measurement.

The tests were carried out in February 2018 inside four Tinkwino tomato greenhouse, each one an area of 10,000 m^2^ whose production is marketed in the European market. It is located in the Cañada de San Urbano, province of Almería, autonomous community of Andalusia in Spain. The distribution of the plantation is the classic one for a greenhouse of tomato, with corridors of 1.2 m (Figure 3B), 50 cm of distance between plants (Figure 3C), separation between paired lines and ends of the foliages 60 and 100 cm respectively (Figure 3D), and with a length of the main hall being 100 m (Figure 3E).

### 2.11. Field Tests

**Figure 4:**
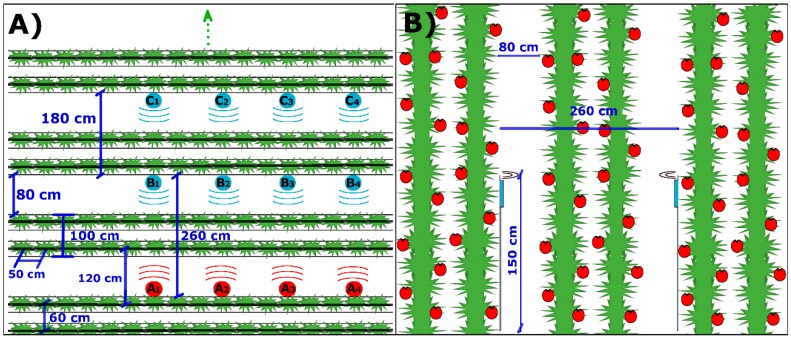
Tomato greenhouse diagram and WSN deployment layout in the tests. (**A**) View from above, (**B**) transverse view.

The WSN network was deployed in the greenhouse following the distribution of Figure 4A,B. The mast that supports each node has a base of 17 kg to give stability and avoid possible rocking. The sink node (red color) and sensor (light blue) were initially in positions A1 and B1 respectively at the same height. The data sink with RSSI information in dBm were sent every 10 s for 10 min, arrived at the sink node. After completing the RSSI data collection, these were moved 2 m to the right (A2, A3, A4 and B2, B3, B4), and the average in the four positions was recorded. This process was repeated again at a different height. Later the sensor node moved from position Bx to Cx and so on as long as there was connectivity with the sink Ax located at the end of the greenhouse lacking line of sight. Finally, we elaborated the measurement curve and contrasted it with the propagation models of radio waves in the presence of vegetation.

In the literature reviewed, transceiver transmission power and antenna gains were assumed to be those in the technical sheets, and these data were used to calculate the propagation loss later. However, these were far from real and altered the accuracy of subsequent estimates. For this reason, through field tests, the real value of the transmit power was measured so that the results were reliable.

## 3. Results and Discussions

**Figure 5:**
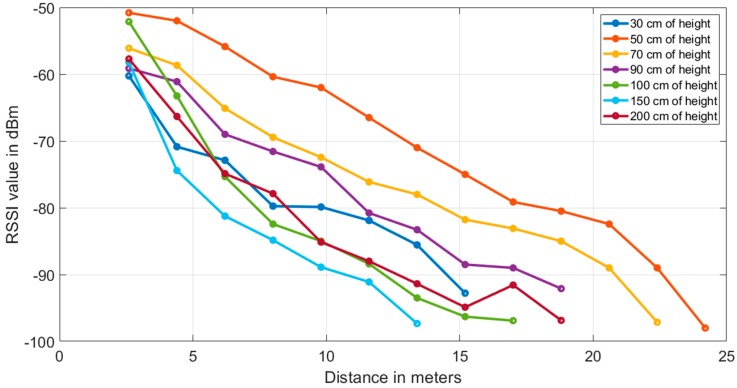
Signal level in dBm and maximum coverage between the transmitting node (Tx) and receiver (Rx) node at different heights with respect to the ground (30 cm, 50 cm, 70 cm, 90 cm, 100 cm, 150 cm, and 200 cm).

During the field-tests, omnidirectional antennas were used in each node because they were traditionally used on agricultural deployments for WSN devices for the purpose of cover links P2MP (point-to-multipoint communication) and MP2MP (multi-point to multi-point) in a real solution. The height of the transmitter and receiver omnidirectional antennas during the tests carried out inside the greenhouse were 30 cm, 50 cm, 70 cm, 90 cm, 100 cm, 150 cm, and 200 cm from the ground. Based on the power levels recorded at the receiving node, the largest and smallest measured ranges were when the antenna height of the transmitting node (Tx) and receiver (Rx) were both 0.5 m and 1.5 m respectively using the 2.4 GHz band (Figure 5).

Table 2 compares the attenuation values in the greenhouse (empirical PL) with respect to the separation distance of the nodes Tx and Rx measured in meters, when they were located 0.5 m and 1.5 m above the ground. The separations between the nodes were initially 2.6 m and then increased steadily by 1.8 m due to the plantation frame that has the scheme of Figure 4. The variations of the values in the attenuations were due to the conformation in the structure of the vegetation different heights. On average, in our case study, the highest density in vegetation consisting of stems, leaves and fruits was at a height of 1.5 m.

**Figure 6:**
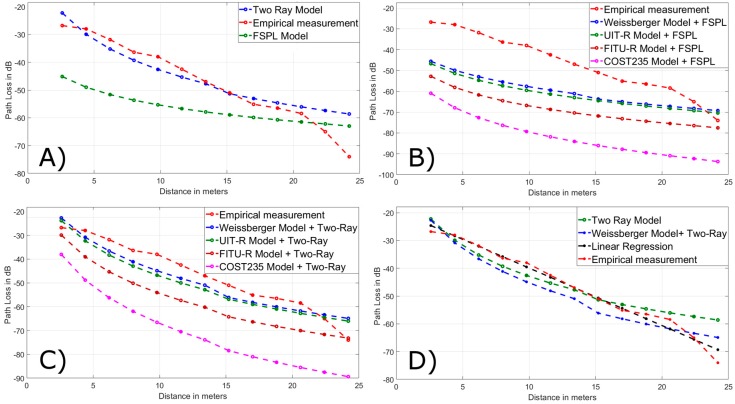
Field measurement at 0.5 m of soil vs. (**A**) models with line of sight (LOS), (**B**) empirical models + FSPL, (**C**) empirical models + two-ray, (**D**) optimized model (linear regression) and the others closest in values.

**Figure 7:**
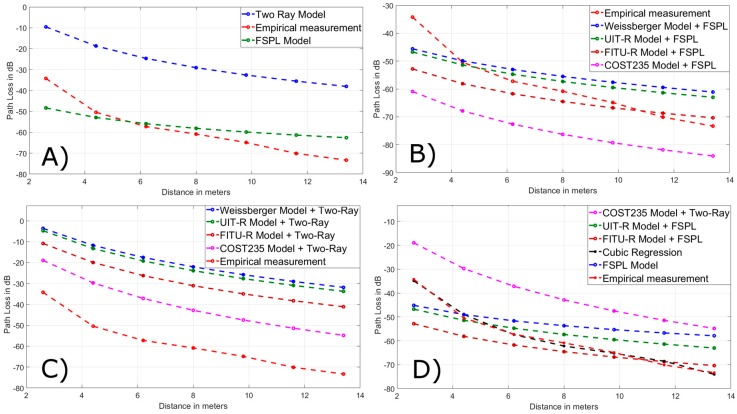
Field measurement at 1.5 m of soil vs. (**A**) models with LOS (**B**), empirical models + FSPL (**C**), empirical models + two-ray, (**D**) optimized model (cubic regression) and the others closest in values.

With the Tx and Rx nodes at 0.5 m from the ground, the results obtained in the field tests were compared with the models with LOS (Line of Sight), the FSPL (Free-Space Path Loss) and two-ray with the graphs of curves shown in Figure 6A. In addition, the COST235, FITU-R, ITU-R, and Weissberger models were added to the losses of the unobstructed path corresponding to the corridors and spaces between floors with the FSPL and two-ray models in 6B and 6C respectively. From all these graphs it was concluded that the closest models were two-ray and Weissberger, adding the attenuation of two-ray. Analogously it is the analysis in Figure 7A–C working with the nodes Tx and Rx at 1.5 m above the ground.

### New Optimized Model with Verification of Values

**Figure 8:**
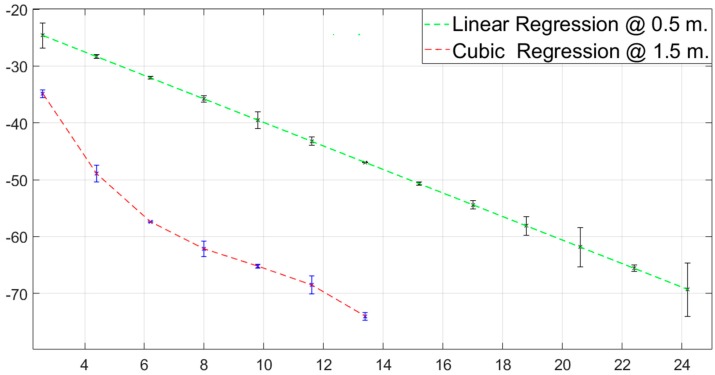
Linear and cubic regression for measurement at 0.5 m and 1.5 m respectively along with its deviation.

The Matlab program (R2018a) has been used to develop our optimized model through linear regression when the nodes were at 0.5 m from the ground. The mathematical equation used was: *y* = −2.0685d − 19.252 (where *d* is the distance between the nodes T_x_ and R_x_). If the nodes T_x_ and R_x_ are placed at a 1.5m distance from the ground, the mathematical equation of the optimized model was obtained by cubic regression using the same software is this time: *y* = −0.056156*d*^3^ + 1.6125*d*^2^ − 17.006*d* − 0.56299. The Figure 6D and Figure 7D show the best precision of our models with respect to the others. The other models with the closest approximation of values were two-ray and Weissberger a 0.5 m and COST235, UIT-R, FITU-R, FSPL for 1.5 m. Likewise, the percentage of error (% error) was found with the following formula: Abs{[1 − (X_i empirical_/X_i model_)] × 100%}, where X_i_ is the measured value (X_i empirical_) or predicted (X_i model_) in a specific distance. In our models, they were less than 9%, better than the other models, and are corroborated in Figure 8, with the variability of each proposed model in relation to empirical measurement values. The Table 3 and Table 4 in the shaded records validate the optimization of the equations.

Regardless of the brand or model of the sensor node, with the developed models it is possible to plan the maximum distance that two nodes can be separated knowing the reception sensitivity and the EIRP (effective isotropic radiated power) and from there if you want to expand the coverage you could do it with a multi-hop topology. For example, the signal sent from the transmitter node was attenuated through the vegetation, and was detected in the receiver with a strength of −87 dBm, being the link margin of 10 dB because the receiver sensitivity of the node was −97 dBm in our case [−87 dBm − (−97 dBm) = 10 dB]. There is acceptable link stability from a link margin equal to or greater than 10 dB, according [75,76,77,78,79]. With these described features, it is possible to link two nodes at a maximum distance of 20.6 and 13.6 m if they are at a height of 0.5 or 1.5 m, respectively. In this same example, maintaining the positions of the transmitter and receiver with other features of nodes (different brand and model), if the receiver detects −70 dBm, and the receiver sensitivity of the new node was −90 dBm, then, likewise, the distance between the two nodes could be increased until a link margin of 10 dB. Therefore, the contribution of our model is useful for the prediction of the distance between two nodes and the planning of nodes deployment.

## 4. Conclusions

In the present work, a complete study of the propagation models for tomatoes greenhouses was carried out. The characteristics of tomato plantation caused an attenuation due to vegetation, and to the height, so by means of this study it has been possible to see if the theoretical propagation models adjust well to the conditions that exist in the plantations of tomatoes. With the novel mathematical models of propagation of radio waves developed, a better estimation of its attenuation can be obtained for this specific environment. In this way, the results of this study allow planning the deployment of WSN in terms of maximum distances between nodes and consequently in the number of nodes that would be used.

It is recommended to place the omnidirectional antenna at a height to half a meter above ground level because greater coverage between two nodes in the deployment can be reached. By optimizing the number of nodes required to monitoring and/or automatizing the greenhouse crop, we could reduce the cost of investment, redistributing the economic savings to reinvest it on their crops. With this height, the classical models diverge up to an error percentage of 42.84%, with the Weissberger and two-rays models being the closest empirical measurement after our proposed model.

The lowest coverage was obtained at 1.5 m, and the models that most closely approximate the empirical values were those of ITU-R, ITU-R, and FSPL that can diverge on average up to an error rate of 30%. The two models that we have developed have percentages of error lower than 9% when the nodes are at a height of less than one and a half meters, and less than 3% with a half meter of separation with the ground.

Our models are replicable and help more accurately to plan, predict coverage, and optimize the number of nodes in a network of wireless sensors operating in the 2.4 GHz band in a tomato greenhouse. Future work could examine how to generalize the model to other types of greenhouse crops. However, new tests should be carried out for other crops and different greenhouse conditions, for example, with different types of planting frames.

## Figures and Tables

**Table 1:** Empirical propagation models.

Model	Equation
The modified exponential decay model of Weissberger	L_Weiss_ = 0.45*f^0.284^d,* 0 m < *d* < 14 m L_Weiss_ = 1.33*f^0.284^d^0.558^*, 14 m < *d* < 400 m The frequency *f* in GHz and the depth of the trees, *d*, in meters. Applicable at frequencies of 0.23–95 GHz.
Loss factor of the ITU-R model	L_ITU-R_ = 0.2*f*^0.3^ *d*^0.6^, *d* < 400 m. The frequency *f* in MHz and the depth of the trees, *d*, in meters. Applicable to the frequency of 0.2–95 GHz.
Loss factor of the COST235 model	L_COST235_ = 26.6*f^−0.2^d^0.5^out-of-leaf* L_COST235_ = 15.6*f^−0.009^d^0.26^in-leaf* *f* is the transmission frequency (MHz), *d* is the depth of the trees in meters. Applicable to the frequency of 0.2–95 GHz
FITU-R	L_FITU-R_ = 0.37f*^−018^d^0.59^out-of-leaf* L_FITU-R_ = 0.39*f^−0.39^d^0.25^in-leaf* *f* is the frequency in MHz and *d* is the tree depth in meter, based on millimeter VHF wave measurement data on a short foliage depth (maximum of 400 m)

**Table 2:** Total attenuation of the propagation of the radio wave in the presence of vegetation with nodes at 0.5 m and 1.5 m from the ground.

Model	Distance (m)												
	2.6	4.4	6.2	8	9.8	11.6	13.4	15.2	17.0	18.8	20.6	22.4	24.2
Empirical PL (dB) at 0.5 m	26.8	28	31.86	36.38	38	42.5	47	51	55.13	56.5	58.44	65	74
Empirical PL (dB) at 1.5 m	34.22	50.43	57.25	60.85	64.89	70.11	73.33	-	-	-	-	-	-

**Table 3:** Percentage of error in the 2.4 GHz band with propagation models in the presence of vegetation when the Tx and Rx nodes were 0.5 m above the ground.

% Error	Distance (m)												
	2.6	4.4	6.2	8	9.8	11.6	13.4	15.2	17.0	18.8	20.6	22.4	24.2
FSPL	40.64	42.84	38.30	32.21	31.30	25.04	18.81	13.46	7.92	6.94	4.95	4.52	17.52
Two-Ray	20.44	6.44	9.57	7.41	10.75	6.27	1.52	0.65	3.91	3.44	4.25	13.27	26.25
Weissberger + Two Ray	17.99	9.24	12.99	11.56	15.34	11.66	7.76	9.15	5.25	5.93	5.43	2.51	13.99
Linear Regression	8.81	1.25	0.68	1.62	3.85	1.73	0.06	0.61	1.31	2.82	5.53	0.89	6.77

**Table 4:** Percentage of error in the 2.4 GHz band with propagation models in the presence of vegetation when the Tx and Rx nodes were 1.5 m above the ground.

% Error	Distance (m)						
	2.6	4.4	6.2	8	9.8	11.6	13.4
FSPL	29.22	4.69	2.43	4.72	8.39	14.31	17.17
Two-Ray	258.13	169.76	132.23	109.25	99.02	97.30	92.77
COST235 + 2-Ray	80.52	69.52	54.08	42.02	36.65	36.36	33.75
UIT-R + FSPL	26.79	1.9	4.62	6.13	9.05	14.24	16.37
FITU-R + FSPL	35.22	13.22	7.28	5.69	2.85	2.04	4.20
Cubic Regression	1.85	3.01	0.26	2.11	0.49	2.34	0.93
